# PvDBP gene amplification is associated with functional immune evasion by Plasmodium vivax in vivo

**DOI:** 10.21203/rs.3.rs-9081241/v1

**Published:** 2026-04-07

**Authors:** Léa Baldor, Dynang Seng, Sokleap Heng, Nimol Khim, Sreyneat Hor, Sopheany Thin, Nichole Salinas, Niraj Tolia, Chetan Chitnis, Ivo Mueller, Siavash Foroughi, Christopher King, Eugenia Lo, Benoit Witkowski, Claude Flamand, Lionel Feufack-Donfack, Jean Popovici

**Affiliations:** Institut Pasteur du Cambodge; Institut Pasteur du Cambodge; Institut Pasteur du Cambodge; Institut Pasteur du Cambodge; Institut Pasteur du Cambodge; Institut Pasteur du Cambodge; National Institute of Allergy and Infectious Disease; National Institute of Allergy and Infectious Diseases, National Institutes of Health; Institut Pasteur; Walter and Eliza Hall Institute of Medical Research; The Walter and Eliza Hall Institute of Medical Research; Center for Global Health and Diseases, Case Western Reserve University, Cleveland, Ohio, USA 44106; Drexel University; Pasteur Institute of Cambodia; Institut Pasteur du Cambodge; Institut Pasteur du Cambodge; Institut Pasteur du Cambodge

## Abstract

The key ligand involved in *Plasmodium vivax* reticulocyte invasion is the Duffy Binding Protein (PvDBP) which binds the Duffy receptor on reticulocytes. Anti-PvDBP human monoclonal antibodies can inhibit PvDBP-Duffy receptor binding and neutralize reticulocytes invasion *in vitro*. However, parasites with multiple copies of the *pvdbp* gene are protected *in vitro* against neutralization.

Here, we evaluated whether this gene amplification also protects parasites *in vivo.*We hypothesized that: (i) multi-*pvdbp* copy parasites are more frequent in areas with a high *P. vivax* prevalence, (ii) individuals with naturally acquired binding inhibitory anti-PvDBP Abs (BIAbs) are predominantly infected over time by multi-copy parasites and (iii) multi-copy parasites infect asymptomatic carriers more frequently than symptomatic individuals.

We analyzed samples from a 2019–2020 longitudinal cohort of individuals living in nine villages in Eastern Cambodia with low (~5%) to high (~30%) *P. vivax* prevalence. Using a PCR assay targeting the boundaries of the *pvdbp* duplication, we estimated the frequency of multi-copy parasites over time. Then, using a flow cytometry assay, we determined the presence of naturally acquired BIAbs in 657 participants’ plasma. Finally, we compared parasite gene copy number over the 21-month follow-up in cohort participants according to the presence of BIAbs at the start of the study. In parallel, we determined the frequency of *pvdbp*duplication in samples collected among symptomatic treatment-seeking patients in the same area over the same period. We compared the frequency of infection with multi-copy parasites between asymptomatic cohort members and symptomatic patients.

We found a significant association between *P. vivax* prevalence and the proportion of multi-copy parasites, which ranged from 35% in low prevalence villages to 47% in high prevalence villages (p=0.0246). We also observed that the more inhibitory the Abs in the hosts’ plasma, the higher the proportion of multi-copy parasites: 87% (40/46) from individuals with high BIAbs while 38% (193/514) from individuals without any BIAbs (p<0.0001). This association between immunity and infection by multi-copy parasites remained consistent over the 21-month longitudinal follow-up. Finally, we found that the frequency of multi-copy parasites was higher in asymptomatic carriers than in symptomatic individuals.

Overall, these results indicate that *pvdbp* duplication helps the parasites to avoid the hosts’ anti-PvDBP immunity *in vivo*. It warrants further investigations to determine if immunization with a PvDBP vaccine could overcome this immune evasion mechanism.

## Introduction

*Plasmodium vivax* has the widest geographic distribution of the human infective *Plasmodium* species, and more than three billion people live within the transmission limits of *P. vivax* ^1^. Considered as benign for decades, it is now clear that *P. vivax* malaria is a significant cause of morbidity and mortality in endemic populations ^2,3^. While the prevalence of *P. falciparum* malaria has decreased worldwide, the relative proportion of malaria attributable to *P. vivax* is increasing, including in Cambodia ^4,5^. Unlike *P. falciparum*, *P. vivax* forms a dormant liver stage (hypnozoites) capable of causing multiple recurring blood stage infections (relapses) following a single mosquito inoculation, complicating *vivax* malaria elimination efforts ^6,7^. In addition, our inability to continuously cultivate *P. vivax in vitro* (a major difference with *P. falciparum* which is easily cultivable) ^8,9^, has impeded research efforts to develop *P. vivax* specific interventions. It is therefore crucial to identify strategies beyond conventional control tools to achieve malaria elimination in regions where *P. vivax* predominates ^10^*.*

A vaccine protecting against *P. vivax* infections would be a major asset to eliminate this parasite. The leading *P. vivax* blood-stage vaccine candidate is the *P. vivax* Duffy Binding Protein (PvDBP) and has successfully completed phase I and IIa clinical trials ^11–13^. This parasite ligand binds the Duffy Antigen Receptor for Chemokines (DARC, also called Duffy) on the surface of red blood cells (RBCs). This critical interaction enables the process of invasion ^11,12,14,15^. Individuals lacking the Duffy receptor are almost completely protected against *P. vivax* infections ^16^, and recent studies suggest that infection of Duffy negative individuals may involve the production of low amounts of Duffy receptor in young reticulocytes ^17,18^, highlighting the critical role of the PvDBP-Duffy binding for the parasite invasion.

One of the major obstacles to develop malaria blood-stage vaccines is the high polymorphism of the parasitic antigens. For *P. vivax*, the PvDBP region II (PvDBPII), binding to the Duffy receptor, is well characterized. PvDBPII comprises many highly polymorphic and often immune-dominant epitopes ^19–22^.^.^ Most patients infected by *P. vivax* develop antibodies against the DBPII non-binding immuno-dominant epitopes. Only a minority (~10%) develop binding inhibitory antibodies targeting the relatively conserved amino acids necessary for the Duffy receptor binding ^23–25^. Those individuals able to produce such binding inhibitory antibodies (BIAbs) are at lower risk of subsequent *P. vivax* infection ^23,25^.

However, we have previously shown that parasites harboring multiple copies of the gene encoding PvDBP may bypass the *in vitro* neutralization by BIAbs, regardless of their sequence polymorphism ^26^. It is therefore critical to determine whether *pvdbp* amplification operates as a functional immune evasion mechanism in natural infections, potentially undermining vaccine-induced immunity. Here, we retrospectively analyzed data from a longitudinal cohort of individuals living in malaria endemic areas of Cambodia to assess whether *pvdbp* amplification could constitute an immune evasion mechanism and allow *P. vivax* protection against host natural anti-PvDBP immunity *in vivo*.

## Results

A total of 950 individuals living in 391 households were enrolled in a longitudinal cohort and followed monthly over 21 months. Participants were 53% female (508/950) and 47% male (442/950). The median age was 19·5 years (IQR 12–32; range 5–66). Village sizes ranged from 8 to 245 participants, with the largest contributions from Sraektum (*n* = 245), Sraepreas (*n* = 137), and Sraeampilkroam (*n* = 127).

Across the 21-month schedule, *pvdbp* copy number calls were available for 127 individuals at baseline and 40 to 82 individuals per subsequent time point, totaling 997 interpretable measurements over the duration of follow-up (Appendix 1).

We first screened baseline plasma samples (M0) from a subset of cohort participants for naturally acquired anti-PvDBP BIAbs. In total, 657 of the 950 enrolled individuals (69%) were included. Selection was conducted at the village level. All participants who experienced at least one *P. vivax* infection at any point during the longitudinal follow-up were included, except for nine individuals for whom baseline plasma samples were unavailable (*n* = 241). In addition, 416 participants for whom plasma samples were available but whom never had a *P. vivax* infection detected during the study period were included. These uninfected individuals were distributed across villages to ensure that at least 35% of participants per village were represented (range: 35%–100%). Among them, priority was given to participants with ≥6 follow-up time points (*n* = 355). Individuals with fewer follow-up time points and no detected *P. vivax* infection were included only in villages with low recruitment numbers to increase the sample size.

**Figure 2:**
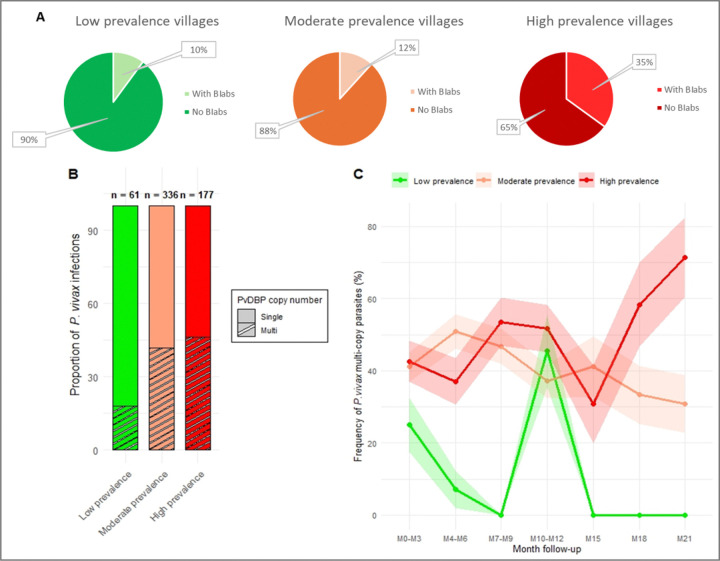
Association between *P. vivax* prevalence and (i) presence of BIAbs in patients’ plasma, (ii) infection by multi-copy parasites. Proportion of individuals with anti-PvDBP BIAbs in low (left panel), moderate (middle panel) and high (right panel) *P. vivax* prevalence villages (**A**). Association between the prevalence at M0 and the proportion of infection by multi-copy (hatched) or single copy (empty) parasites (pooled data) (**B**). Correlation between the frequency of multi-copy *P. vivax* parasites and *P. vivax* prevalence during the 21-month cohort follow-up (**C**).

Of the 657 baseline samples, 15·5% (102/657) had detectable anti-PvDBP BIAbs (defined as > 90% binding inhibition at 1:5 dilution). The proportion of individuals with BIAbs differed significantly across prevalence strata (Chi-square test, P=0·0011), being highest in high-prevalence villages (35%, 42/120) compared to moderate (12%, 41/349) and low prevalence areas (10%, 19/188) (Fig. 2A).

Among the same individuals, we assessed infection by *pvdbp* multi-copy parasites at each time-point during the longitudinal follow-up using a qualitative PCR (single copy or more than 1 copy). 574 *P. vivax* positive infections coming from 8 different villages gave interpretable results. Over the 21 months, we found 233 (40·6%) isolates infected with duplicated *pvdbp* and 341 (59·4%) presenting single *pvdbp* copy. The frequency of multi-copy isolates increased in villages with high *P. vivax* prevalence (47% of isolates) compared to low prevalence areas (35% of isolates) (Kruskal-Wallis and Dunn’s correction, P=0·0246) (Fig. 2B). This association remained consistent throughout the 21 months of follow-up (Fig. 2C).

**Figure 3:**
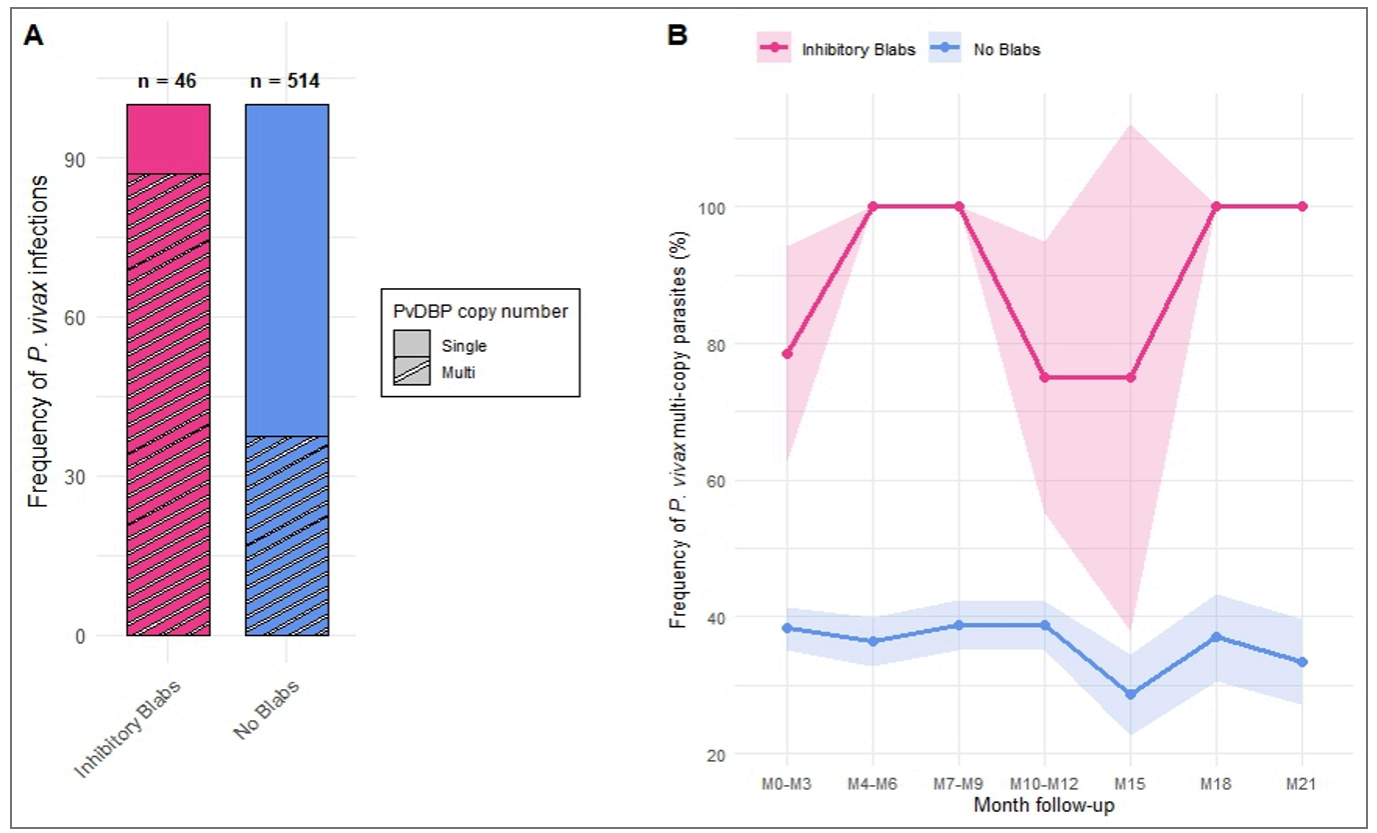
Association between anti-PvDBP inhibitory immunity and multi-copy parasite infection dynamics over time. Association between the potency of PvDBP-Duffy BIAbs in participants (pooled data) and the proportion of infection by *P. vivax* multi-copy (hatched) or single copy parasites (empty) **(A).** Longitudinal analysis of the association between the presence of inhibitory BIAbs in participants’ plasma and the frequency of infection by *pvdbp* multi-copy parasites (cumulated data, *n*=560) **(B).**

We then tested if multi-copy parasites predominantly infected hosts displaying BIAbs. Pooling infection-level data (each PCR-confirmed *P. vivax* infection at a given time point) across participants, we compared the frequency of multi-copy *P. vivax* infections among individuals with or without highly inhibitory BIAbs (>90% PvDBP-DARC inhibition) at baseline. We observed that 87% (40/46) of parasites infecting participants presenting highly inhibitory antibodies carried *pvdbp* amplification, while only 37·3% (192/514) were infecting individuals without BIAbs (Fisher test, P<0·001, *n*=613, OR 11.19, [95% CI 4·51–27·77]) (Fig.3A) showing that the more inhibitory the antibodies in the host plasma, the higher the proportion of multi-copy parasites.

To account for potential cofounders and repeated infections within individuals, we further assessed the association between (i) the presence of BIAbs, (ii) the village prevalence class (low, moderate, high), (iii) the sex, (iv) the age of the participants and the likelihood of infection by multi-copy parasites using GAMs. In multivariate analyses, inhibitory status emerged as the strongest predictor of infection with multi-copy parasites (OR 11·19, 95% CI 4·51–27·77; p<0·001) (Table 1). Village prevalence class was also significantly associated with multi-copy parasites infection, with higher odds in moderate-prevalence villages (OR 2·54, 95% CI 1·22–5·31; p=0·013) and high-prevalence villages (OR 2·39, 95% CI 1·07–5·34; p=0·033), compared with low-prevalence villages (Table 1). No significant association was observed between males and females (OR 1·14, 95% CI 0·69–1·89; p=0·612) (Table 1). Age showed a non-linear trend that did not reach conventional statistical significance (edf=5·96, p=0·070) (Table 1). After adjustment, inhibitory antibody status and village-level transmission intensity remained the primary determinants of *pvdbp* multi-copy infections, independent of sex, age, and temporal variations.

We then compared the frequency of infection by multi-copy parasites over time between individuals without anti-PvDBP BIAbs and individuals with highly inhibitory BIAbs. We observed that for individuals with no BIAbs, the frequency of infection with multi-copy parasites remains around 40% from month 0 to month 21 (Fig.3B). However, individuals with highly inhibitory BIAbs are predominantly infected by multi-copy parasites (70–100% of *P. vivax* infections with multi-copy parasites), and this association lasts for several months (Fisher test, P = 0·01936–0·2174 depending on follow-up month, *n*=560) (Fig.3B).

Finally, we compared the prevalence of multi-copy parasites between these asymptomatic infections identified in the longitudinal follow-up and those from symptomatic cases collected in the same study area during the same study period. Among the asymptomatic individuals, 41% (235/574) of *P. vivax* parasites carried the *pvdbp* amplification, whereas only 23% (46/203) were multi-copy parasites in symptomatic cases (Fisher test, P<0·0001) (appendix 2).

## Discussion

Previous studies have well described gene amplifications in *Plasmodium* parasites as a mechanism of drug resistance. For instance, in *Plasmodium falciparum*, the copy number variation of *pfmdr1* and *pfplasmepsin2* are associated with parasite resistance to mefloquine and piperaquine, respectively ^29,30^. Gene copy number variation is also observed in *Leishmania* parasites with evidence that amplification can lead to phenotypic adaptations in response to environmental changes ^31^ and is associated with drug resistance and tissue tropism ^32^. In *P. vivax*, gene amplification has been shown to be associated with evasion from the host’s immune response *in vitro* ^26^, but evidence from *in vivo* human infections has been lacking.

We show here that, in a Cambodian endemic area, higher *P. vivax* prevalence is associated with a greater proportion of individuals who develop naturally acquired anti-PvDBP BIAbs, as well as higher frequency of parasites carrying *pvdbp* gene amplification. Individuals displaying BIAbs are also consistently infected by multi-copy parasites, and this association persists for several months. This raises the possibility that implementing a vaccine targeting protective binding epitopes of PvDBP could select for parasites carrying *pvdbp* gene amplification.

Although our study supports an association between *pvdbp* gene amplification and immune evasion in *P. vivax* infection, the molecular mechanisms leading to this resistance remain unknown. We have previously demonstrated that *pvdbp* gene amplification was associated with increased mRNA expression level ^26^. However, we still do not know to what extent it affects the PvDBP invasion pathway and potentially contributes to immune evasion.

We have previously demonstrated that increasing anti-PvDBP concentrations up to 1000 μg/mL can neutralize invasion by multi-copy parasites *in vitro* ^26^*.* Here, anti-PvDBP BIAbs titers were not quantified. It is therefore possible that these naturally acquired antibody levels were insufficient to fully block invasion by parasites with *pvdbp* amplification, while vaccination might induce higher antibody titers capable of neutralizing invasion by those multi-copy parasites. Conversely, naturally exposed individuals can acquire high titers of BIAbs and these antibody levels remain stable for several years ^23^. Future work should examine whether vaccination regimens could induce sufficiently high and durable BIAbs titers to block invasion by multi-copy parasites *in vivo*.

Other proteins involved in the invasion pathway have also been described. It has recently been shown that targeting AMA1 prevents sporozoite invasion into hepatocytes *in vitro* and reticulocyte invasion by merozoite *in vitro* ^33^. To effectively neutralize these amplified parasites, combining approaches targeting DBP and other *P. vivax* critical invasion ligands, such as AMA1, should then be considered.

The potential clinical impacts of infection with multi-copy parasites also remain poorly understood. It might be linked with differences in parasite density, parasite total biomass, or symptomatology among infected individuals. Here we show that multi-copy infections are more frequently found in asymptomatic than symptomatic individuals. This is consistent with a potential role of *pvdbp* gene amplification as an immune evasion mechanism, as strong immunity might prevent the onset of symptoms or reduce their severity, leading to asymptomatic carriers. Multi-copy parasites, able to bypass this immunity, might then be selected and more prevalent in asymptomatic than symptomatic carriers. However, opposite findings were observed in Ethiopia. In a study conducted in 2019, isolates carrying multiple *pvdbp* copies were predominantly found in symptomatic patients compared to those with asymptomatic infections ^34^. This observed difference could result from biological differences between the two countries in the function of PvDBP gene duplication or, alternatively, could result from epidemiological differences as in Ethiopia, malaria transmission is peri-domestic with widespread exposure across the population, while in Cambodia, only individuals engaging in forest activities are at high risk of malaria. Additional studies in different endemic areas are needed to understand the host-parasite interactions driving the selection of *pvdbp* amplification.

This study has some limitations. First, the prevalence of the disease decreased substantially between the beginning and the end of the cohort study period because of intensified malaria elimination efforts by Cambodian authorities. As a result, even when targeting the highest transmission areas, our sample size remained relatively small. Second, our study was conducted in a single endemic country. Investigating other *P. vivax* endemic regions, with different transmission and prevalence settings would allow for comparison of how multi-copy parasites persist under varying epidemiological conditions and enable better understanding of the extent of this *pvdbp* immune evasion mechanism. Finally, our study was conducted over a period of only 21 months. Extending it over multiple years would provide a clearer understanding of the dynamics of *pvdbp* amplification under repeated exposures or changes in endemicity, as well as its association with host immunity.

In conclusion, our findings show that in Cambodia, *pvdbp* amplification is likely associated with the ability of *P. vivax* parasites to evade naturally acquired anti-PvDBP immunity *in vivo*, raising concerns about the efficacy of vaccine strategies targeting PvDBP. Further research is needed to determine the extent to which PvDBP-based immunization could overcome this newly described evasion mechanism.

## Methods

### Study design and participants

We analyzed blood and plasma samples collected from individuals enrolled in a longitudinal cohort conducted in the Keo Seima district, Mondulkiri province (described in ^27^).

A total of 950 participants aged 5 years or older who had lived in the 10 selected villages for more than 3 months were enrolled. They were monitored monthly from January 2019 to March 2020, with three additional follow-up points between June and December 2020. Individuals with chronic illnesses or disabilities that could hinder participation or communication were excluded. In parallel, blood samples from treatment-seeking *P. vivax* infected patients visiting health centers in the same area during the same study period were collected and included as symptomatic cases in this analysis.

This study was approved by the National Ethics Committee for Health Research, Ministry of Health, Kingdom of Cambodia (references 239-NECHR and 057-NECHR) and by the Institut Pasteur Institutional Review Board (Reference 2017–03). All participants or their parents or legal guardians were required to fill and sign a written consent form, and assent was obtained from children aged 12 and over.

### Procedures

At each collection time point, capillary blood was collected in EDTA microtainer. Plasma and RBC pellet were then separated. DNA was extracted from the pellet and used for PCR diagnostic. Infection by *Plasmodium spp.* parasites were determined using species-specific real-time PCR as previously described ^28^.

For symptomatic cases, a rapid diagnostic test was performed. If positive, capillary blood was collected in EDTA microtainer. PCR diagnostic and species screening were performed as described above. Thin and thick blood smears were also examined by microscopy to confirm infection and parasitemia.

For *pvdbp* copy number determination, two different PCR approaches were used. For low parasitemia infections, we used a semi-nested PCR targeting the boundaries of the *pvdbp* duplication, as previously described ^26^. For symptomatic carriers, a qPCR using *P. vivax* tubulin gene as a single-copy reference was performed, as previously described ^26^. These complementary approaches were selected based on parasitemia levels and both specifically detect the presence of *pvdbp* duplication as previously validated.

For DBPII-erythrocyte binding assays, we used a flow-cytometry based assay previously published^26^. Cohort participant plasma was used at a final dilution of 1 in 5 in the presence of recombinant DBPII protein (final concentration 0.8 μg/mL). Rabbit polyclonal anti-PvDBPII was diluted at 1 in 10000. Goat anti-rabbit IgG (H+L) Alexa Fluor 488 conjugated antibodies at dilution 1 in 500 was used for flow cytometry detection. Negative binding inhibitor control was made of plasma from malaria-naïve donors while positive inhibitor control was made of naïve plasma spiked with either the 092096 or 99100 human monoclonal antibodies (humabs) at 2ug/mL final concentration ^26^. All binding values were normalized to a no-plasma control. Plasmas were considered as containing BIabs if more than 90% binding inhibition compared to no-plasma controls were measured. Data were analyzed using FlowJo software v.10 (Tree Star, Inc).

### Outcomes

This study is based on a retrospective analysis of samples and date collected from a longitudinal cohort conducted in 2019–2020 in Mondulkiri province, Cambodia. The outcomes of this work were to (i) examine the association between village-level *P. vivax* prevalence and the frequency of multi-copy parasites, (ii) assess the relationship between the infection by multi-copy parasites and the presence of highly inhibitory anti-PvDBP antibodies in individuals’ plasma, (iii) evaluate the persistence of these associations over time and (iv) determine the frequency of infection by multi-copy parasites in asymptomatic and symptomatic individuals.

### Statistical analysis

Analyses were conducted at different levels depending on the research question. Village-level analyses were based on aggregated infection data. Individual and infection-level analyses considered each PCR-confirmed *P.vivax* as the unit of observation, with repeated infections within the same individual accounted for in regression models. Villages were classified into low (4–8%), moderate (12–16%) and high (>30%) parasite prevalence groups, as determined at the start of the longitudinal follow-up^27^. The frequency of multi-copy parasites across villages categorized by low, moderate, or high *P. vivax* prevalence was compared using the Kruskall-Wallis test, with Dunn’s post-hoc correction for pairwise comparisons. The correlation between village-level *P. vivax* prevalence and the frequency of duplicated parasites was assessed using Spearman’s rank correlation.

To evaluate the association between the presence of highly inhibitory BIAbs and *P. vivax* prevalence (village-level), and between Blabs and infection by multi-copy parasites (infection-level), Chi-square tests were applied. Fisher’s exact test was used to examine whether the presence of Blabs at baseline (M0) was associated with subsequent infection by multi-copy parasites during follow-up.

To assess the association between the presence of inhibitory antibodies and the likelihood of *P. vivax* parasites carrying multiple copies of the *pvdbp* gene, we also used a generalized additive model (GAM). A logistic regression framework with penalized regression splines was applied (mgcv package in R), allowing flexible adjustments for non-linear effects of continuous covariates and r within-individual correlation due to repeated measurements. The binary outcome was the presence of *pvdbp* multi-copy parasites. The main exposure of interest was participants’ antibody-inhibitory profile (inhibitory vs non-inhibitory). Models were adjusted for sex, age (modelled with cubic regression splines, k=8), and the prevalence class of the village (low, moderate, or high). Follow-up time (months) was incorporated as a spline (k=6) to account for temporal trends. A random-effect term for participant identifier was included to account for repeated measures within individuals. Models were estimated with a binomial family (logit link) using restricted maximum likelihood (REML). Odds ratios (ORs) and 95% confidence intervals (95% CI) were reported for parametric terms, while approximate effective degrees of freedom (edf) and global p values were reported for smooth terms.

Analyses were performed using Graph Pad Prism (V10.01) or R studio (R version 4.3.1). All tests were two-sided and p value < 0.05 was considered statistically significant.

### Role of funding sources

The funders of the study had no role in study design, data collection, data analysis, data interpretation, or writing of the report. The corresponding authors had full access to all the data in the study and had final responsibility for the decision to submit for publication.

## Supplementary Material

Supplementary Files

This is a list of supplementary files associated with this preprint. Click to download.

• Supplementaryappendix.docx

## Figures and Tables

**Figure 1:**
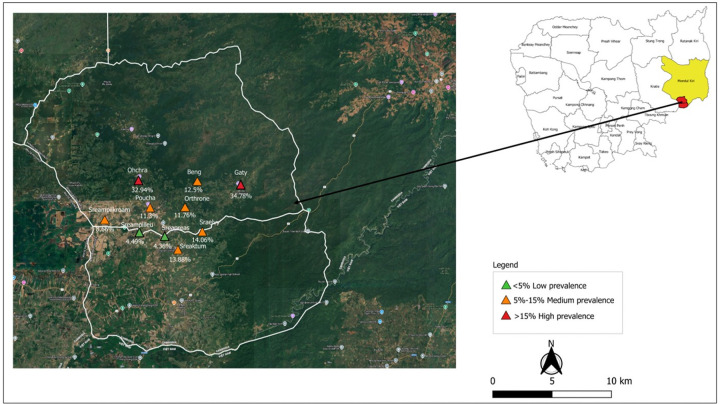
*P. vivax*prevalence in each village at baseline (M0), Keo Seima district, Mondulkiri Province, Cambodia. Red: high prevalence; Orange: moderate prevalence; Green: low prevalence.

**Table 1.** Logistic regression model with individual effects (repeated data) for assessing the likelihood of infection by multi-copy parasites based on binding inhibitory category, village prevalence class (low, moderate, high), sex and age of the participants.

Variable	OR (95% CI)	EDF	p-value

Inhibitory BIAbs (M0)	11.19 (4.51–27.77)		<0.001
Female	1.14 (0.69–1.89)		0.612
Moderate prevalence	2.54 (1.22–5.31)		0.013
High prevalence	2.39 (1.07–5.34)		0.033
Age (years)		5.96	0.070
Follow-up time (month)		1.00	0.510
Participant ID		0.00	0.447

OR: Odds Ratios. 95% CI: 95% Confidence intervals. EDF: Empirical Distribution Function.

## Data Availability

De-identified patient data will be made available upon reasonable request.
